# Neuroprotective effects of vitamin D on high fat diet- and palmitic acid-induced enteric neuronal loss in mice

**DOI:** 10.1186/s12876-018-0905-9

**Published:** 2018-11-21

**Authors:** Sara Larsson, Ulrikke Voss

**Affiliations:** 1 Unit of Molecular Endocrinology, Department of Experimental Medical Science, Lund University, Sölvegatan 19, BMC C11, 22184 Lund, Sweden; 2 Unit of Neurogastroenterology, Department of Experimental Medical Science, Lund University, Sölvegatan 19, BMC B11, 22184 Lund, Sweden

**Keywords:** Enteric nervous system, Vitamin D, High fat diet, Neuronal survival, Gastrointestinal tract, Peroxisome proliferator-activated receptor gamma

## Abstract

**Background:**

The role of vitamin D in obesity and diabetes is debated. Obese and/or diabetic patients have elevated levels of free fatty acids, increased susceptibility to gastrointestinal symptoms and are suggested to have altered vitamin D balance. The enteric nervous system is pivotal in regulating gastrointestinal activity and high fat diet (HFD) has been shown to cause loss of enteric neurons in ileum and colon. This study investigates the effect of vitamin D on HFD- and palmitic acid-induced enteric neuronal loss in vivo and in vitro.

**Methods:**

Mice were fed either a normal diet (ND) or HFD supplemented with varying levels of vitamin D (from 0x to 20x normal vitamin D level) for 19 weeks. Ileum and colon were analyzed for neuronal numbers and remodeling. Primary cultures of myenteric neurons from mouse small intestine were treated with palmitic acid (4x10^-4^M) and/or 1α,25-hydroxy-vitamin D3 (VD, 10^-11^- 10^-7^M) with or without modulators of lipid metabolism and VD pathways. Cultures were analyzed by immunocyto- and histochemical methods.

**Results:**

Vitamin D supplementation had no effect on enteric neuronal survival in the ND group. HFD caused substantial loss of myenteric neurons in ileum and colon. Vitamin D supplementation between 0-2x normal had no effect on HFD-induced neuronal loss. Supplementation with 20x normal, prevented the HFD-induced neuronal loss. In vitro supplementation of VD prevented the palmitic acid-induced neuronal loss. The VD receptor (VDR) was not identified in enteric neurons. Enteric glia expressed the alternative VD receptor, protein disulphide isomerase family A member 3 (PDIA3), but PDIA3 was not found to mediate the VD response in vitro. Inhibition of peroxisome proliferator-activated receptor gamma (PPARγ) and immune neutralization of isocitrate lyase prevented the VD mediated neuroprotection to palmitic acid exposure.

**Conclusions:**

Results show that VD protect enteric neurons against HFD and palmitic acid induced neuronal loss. The mechanism behind is suggested to be through activation of PPARγ leading to improved neuronal peroxisome function and metabolism of neuronal lipid intermediates.

## Background

The role of vitamin D has expanded beyond its classical role in calcium homeostasis. Since animal studies have shown that vitamin D supplementation protects against high fat diet (HFD) induced metabolic parameters [1–3], several efforts have been made to elucidate the role of vitamin D in type-2 diabetes and in obesity, as well as in their complications [4–6]. Recently animal models have likewise shown that various dietary vitamin D supplementations are able to protect against neurodegeneration found in i.e. models of Alzheimer’s disease [7, 8]. While the clinical data on vitamin D supplementation are inconclusive [9, 10], the need for exploring the metabolic potential and cellular mechanisms of vitamin D remains.

Whether produced in the skin through UV radiation or ingested in the diet, vitamin D (25-hydroxy vitamin D3) is in the body converted to its active form 1α,25-hydroxy-vitamin D3 (VD), by the enzyme 1α-hydroxylase also known as cytochrome P450. This enzyme is found mainly in the kidneys but other cells types including immune cells and neurons have been shown to express this enzyme [11, 12]. VD mediates the majority of its effects by binding to, and thereby activating the nuclear VD receptor (VDR). Upon activation, VDR dimerizes with the retinoid-X receptor (RXR) forming a heterodimer, that in turn binds to specific VD response elements (VDREs). [13, 14] However, it is suggested that initiation of VDR mediated responses cannot account for the fast responses seen upon VD stimulation in certain cell types e.g. osteoblasts and intestinal epithelial cells. [15–17] Protein disulphide isomerase family A member 3 (PDIA3), also known as 1,25MAARS, has been suggested as an alternative VD receptor, capable of mediating the fast VD responses and acting as a modulator of cellular function independent of VDR [15, 18]. Besides the receptor mediated VD responses VD, *per se*, has been suggested to act as an antioxidant preventing lipid peroxidation and stabilizing cell membranes [19]. The anti-oxidative potential of VD, independent of either VDR or PDIA3, still needs to be further assessed. In addition, VD is reported to decrease reactive oxygen species (ROS) by increasing levels of the key anti-oxidative enzymes superoxide dismutase (SOD), catalase and glutathione (GSH) [20–22].

Peripheral neuropathy is a common complication of diabetes suggested to be caused by the combination of abnormal blood glucose and lipid levels, oxidative and inflammatory stress and accumulation of advanced glycation products (AGE) [23, 24]. VD deficiency is suggested to be involved in the development of diabetic neuropathy [25]. Further, it is increasingly recognized that obesity and HFD models also are associated with a neurotoxic phenotype. In animals HFD induces anxiety-like behavior and neuronal loss in hippocampus [26, 27], as well as loss of neurons in all parts of the gastrointestinal (GI) tract [28–30]. Clinical data indirectly support these findings as both depression and GI dysfunction are common comorbidities of obesity and the metabolic syndrome [31–33]. The enteric nervous system (ENS), innervates the entire GI tract and is pivotal in the regulation of all GI functions, including motility, secretion and blood flow [34]. The HFD-induced neuronal loss has been suggested to be mediated mainly by the saturated fatty acid palmitic acid (PA) and involve increased oxidative stress and derangement of metabolic pathways [28]. The aim of this study was to investigate effects of VD on HFD-induced neuronal loss and to assess possible pathways by which VD mediates it effects on PA-induced enteric neuronal loss in vitro.

## Methods

### In vivo experiments

C57BL/6J mice (*n*=42, Taconic Bioscience) aged 4 weeks were randomized divided into seven groups (*n*=6 each group), animals were housed in groups. Three groups received normal diet (ND) with different doses of Vitamin D (no vitamin D, ND 0x; normal vitamin D 1.3IU/g [35], ND 1x; twenty times vitamin D, ND 20x). Four groups received high fat diet (HFD) with different doses of vitamin D (no vitamin D, HFD 0x; normal vitamin D, HFD 1x; double vitamin D HFD 2x; twenty times the normal vitamin D, HFD 20x). All diets were purified diets (Research Diets Inc., USA) Table 1 gives nutritional overview and diet details. After 19 weeks mice were sacrificed by cervical dislocation. The GI tract from cardia to rectum was collected, opened along the mesenteric border and emptied. Segments of ileum and transverse colon were fixed and processed for cryo-sectioning [36].

**Table 1:** Nutritional content and vitamin D concentration in the normal diet (ND) and high fat diet (HFD)

	ND/D12450J	HFD/D12492
Protein	20 kcal%	20 kcal%
Carbohydrate	70 kcal%	20 kcal%
Sucrose	6.8 kcal%	6.8 kcal%
Fat	10 kcal%	60 kcal%
Saturated	2 kcal%	19 kcal%
Mono unsaturated	3 kcal%	22 kcal%
Poly unsaturated	5 kcal%	19 kcal%
Vitamin D		
0x	-	-
1x	1.3 IU/g	1.3 IU/g
2x	-	2.6 IU/g
20x	26 IU/g	26 IU/g

### In vitro experiments

#### Primary enteric cultures

C57Bl/6J mice (*n*=46, 20-23g, Janvier Labs, FR) on standard chow diet (R36 Lactamin, SE) were used for in vitro experimentations, animals were housed in groups. Primary cultures of myenteric neurons were prepared from the small intestine. Isolation was done in the morning. Neurons were dissociated using a modification of a previously described method [28, 37]. In brief, anesthetized mice (i.p. injection with Ketalar/Rompun) had their small intestine exposed via midline incisions. The longitudinal muscle layer with attached myenteric ganglia was stripped from approximately 15 cm of the distal small intestine. Tissues were placed in Ca^2+^ and Mg^2+^ free Hank’s balanced salt solution (HBSS) containing 1.9 collagen digestion units (CDU)/mL collagenase 1-A (1.5 mg/mL, Merck, SE) and 4.7 μU/mL protease IX (1.5mg/mL, Sigma-Aldrich, SE) and enzymatically and mechanically separated. Trypsin (0.4 mg/mL, BioChrom AG) and EDTA (0.003%; Merck) were added. Trypsin was inactivated by addition of 50% v/v fetal bovine serum (FBS, Biowest, FR). Cell suspension was centrifuged at 15.6 g for 10 min. Supernatant was removed and pellet washed three times in HBSS, centrifuged at 15.6 g for 10 min and diluted to 0.8 mL in Neurobasal A (NBA) cell culture medium (ThermoFisher Scientific, SE) containing 10% FBS (Biowest, FR), 0.5 mM L-glutamine (K0282, BioChrom AG), 50 U/mL penicillin and 50 μg/mL streptomycin (A2213, BioChrom AG). Cell cultures were prepared by adding 50 μL of the constantly mixed cell suspension into 8-well chambers (30108, SPL, Saveen Werner) prefilled with 450 μL of the NBA cell culture medium. From each animal two 8-well chambers (69mm^2^/well) were prepared. Suspensions from multiple animals were never pooled. Cultures were incubated 4 days prior to experimental treatments.

#### Experimental treatment agents

Stock solutions of palmitic acid (PA, P9767, Merck), VD (D1530, Merck), 7-dihydrocholesterol (7DHC), 16F16 (SML0021 Merck, PDIA3 inhibitor [38]), GW69662 (M6191, Merck, peroxisome proliferator-activated receptor gamma (PPARγ) inhibitor [28]), were prepared, aliquoted and stored at -20 °C. Isocitrate lyase (ICL) antibody (AS10713, Agrisera, SE) and pre-immunization sera (AS03 027, Agrisera, SE) were aliquoted and stored at 4 °C.

#### Experimental set ups

Various sets of experiments were performed. 1. Cultures were exposed to medium containing either PA(4x10^-4^M), VD (10^-11^-10^-7^M), 7DHC (10^-11^-10^-7^M), 16F16 (7x10^-8^-2x10^-5^M), GW69662 (10^-6^M), ICL antibody (1:250) or pre-immunization sera (1:250). 2 Cultures were exposed to either VD (10^-11^-10^-7^M) + PA (4x10^-4^M) or 7DHC (10^-9^-10^-7^M) + PA (4x10^-4^M). 3. Cultures were exposed to either VD (10^-7^M) + experimental treatment agent, PA(4x10^-4^M) + experimental treatment agent or VD (10^-7^M) + PA (4x10^-4^M) + experimental treatment agent. Cultures were incubated 4 days in treatment media with controls run in parallel, fixated in Stefaninins and processed for immunocytochemistry.

#### Histology and immunocytochemistry

For details on primary and secondary antibodies see Table 2. All antibodies were diluted in phosphate buffered saline (PBS) containing 0.25% Triton X-100 and 0.25% BSA (PBS-T-B). For visualization of submucous and myenteric neurons, cryo-sections from ND and HFD mice were immunolabeled with HuC/HuD-biotin and vectastain ABC kit (Vector laboratories Inc., USA), according to manufacturer’s protocol [28, 39]. Morphometric analyses of intestines were on Toluidine blue (0.01% in 60% ethanol for 1.5min) stained cryo-sections.

**Table 2:** Overview of primary and secondary antibodies used in immunocytochemistry

Raised against	Dilution	Code	Source	Host	References
Human neuronal protein, (HuC/HuD)	1:600	A21272	ThermoFisher Scientific, SE	Mouse	[37, 73]
Human gene product 9.5, (PGP 9.5), purified human brain	1:1.200	RA95101	Ultraclone, UK	Rabbit	[73]
PDIA3	1:500	AF8219	R&D systems, USA	sheep	
VDR	1:400	Sc-13133	Kind gift professor BO Nilsson, Lund	Mouse	[74]
S100B (S100), purified bovine brain	1:12.000	Z0311	DAKO, DK	Rabbit	[75]
Alexa Fluor 488 anti-mouse IgG	1:1.000	115-485-166	Jackson Lab Inc, USA	Goat	
Dy-light 594 anti-mouse IgG	1:1.000	715-515-151	Jackson Lab Inc, USA	Donkey	
Alexa Fluor 594 anti- rabbit IgG	1:1.000	711-515-152	Jackson Lab Inc, USA	Donkey	
Alexa Fluor 488 anti goat IgG	1:1000	705-545-147	Jackson Lab Inc, USA	donkey	
Texas Red anti goat IgG	1:400	705-075-147	Jackson Lab Inc, USA	donkey	

Double immunolabeling of cultures were by overnight incubation in moist chamber at 4°C with a mixture of primary antibodies. Secondary antibodies were mixed and incubated 1h at RT. Hoechst (ThermoFisher Scientific, SE) cell nuclei counter staining was performed according to manufacturer’s protocol. Mounting was in PBS:glycerol 1:1 followed by fluorescence microscopy (Olympus BX43, LRI, SE) with appropriate filter setting.

### Analyses

Mucosa and muscularis propria thicknesses were estimated for each mouse using the mean from ten measurements (Aperio ScanScope CS/GL SS5082 and ImageScope). The numbers of HuC/HuD-immunoreactive submucous and myenteric neuronal cell bodies were counted per mm section length. From each mouse, cryo-sections (10μm) cut longitudinally at 3-4 different depths comprising a total length of 30-50mm, were used. Analyses were done blinded to treatment.

Neuronal survival was estimated according to previously described protocol [28, 37]. In brief, neuronal survival after exposure to the various treatments was calculated by counting the total number of HuC/HuD-immunoreactive (IR) neurons in the entire culture well (69 mm^2^) and expressed as percentage of the number of total neurons in the control well run in parallel (% neuronal survival of control). With a minimum of 6 observations in 3 biological repeats, in each treatment group.

Data are presented as means ± SEM and analyzed by GraphPad Prism (GraphPad Software Inc, USA). Statistical significance was determined using two-way analysis of variance followed by Bonferronis post hoc test (in vivo data), or one-way analysis of variance followed by Dunnet’s post hoc test towards controls (in vitro data). A confidence interval of 95% was considered significant.

## Results

### In vivo

**Fig. 1:**
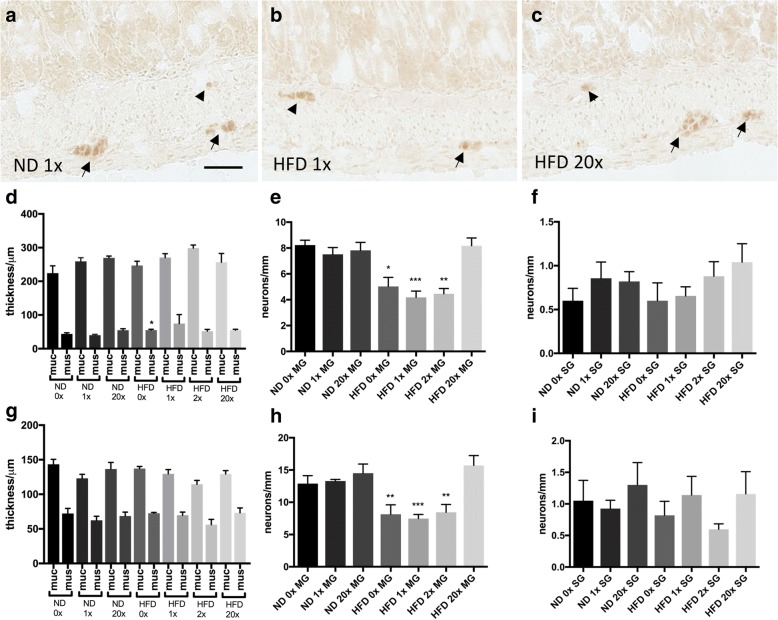
Effects of dietary vitamin D supplementation on high fat diet induced enteric neuronal loss and intestinal morphometric. Representative micrographs (**a**-**c**), morphometrics (**d**, **g**) and numbers of neurons in myenteric (MG) and submucosal (SG) ganglia (**e**, **f**, **h**, **i**) in ileum and colon from mice fed either normal diet (ND) or high fat diet (HFD) supplemented with different concentrations of vitamin D (0 to 20x normal concentration). **a**-**c** cryosections from ileum of ND 1x vitamin D concentration (**a**), HFD 1x vitamin D concentration (**b**) and HFD 20x vitamin D concentration (**c**) immunostained for HuC/HuD-biotin. Myenteric neurons are indicated with arrows and submucosal neurons with arrowheads. **d**, **g** No effect on intestinal morphology is observed in either ileum (**d**) or colon (**g**) in ND or HFD regardless of vitamin D concentration. **e**, **f**, **h**, **i** Numbers of neurons per mm in longitudinal cut sections of the intestine. Vitamin D supplementation (0-20x normal concentration) have no effect on neuronal survival in MG (**e**, **h**) and SG (**f**, **i**) of ileum (**e**, **f**) and colon (**h**, **i**) in ND fed mice. Vitamin D supplementation (0-2x normal concentration) had no effect on HFD induced neuronal loss in MG ileum (**e**) and colon (**h**) but high vitamin D supplementation (20x normal concentration) prevented the HFD-induced myenteric neuronal loss in ileum and colon. Bar represent 20μm

All animals were included in the study. Morphometric analyses of muscularis propria and mucosa in ileum (Fig. 1d) and colon (Fig. 1g) revealed no effect of dietary vitamin D supplementation on intestinal morphology. A slight thickening (p<0.05) of ileum muscularis propria in the HFD 0x group was observed compared to the ND 1x group (Fig. 1d). None of the dietary vitamin D supplementations affected neuronal survival in either ileum (Fig. 1e, f) or colon (Fig. 1h, i) in animals fed the ND. HFD is known to induce loss of myenteric, but not submucosal, enteric neurons in mice [28, 30, 40]. Vitamin D supplementation in the 0-2x range did not attenuate the HFD induced enteric neuronal loss, in ileum (Fig. 1e, f) or colon (Fig. 1h, i). However, animals receiving 20x the normal vitamin D concentration were protected against the HFD-induced neuronal loss in both ileum (Fig. 1e, f) and colon (Fig. 1h, i).

### In vitro, VD effects on primary enteric cultures

**Fig. 2:**
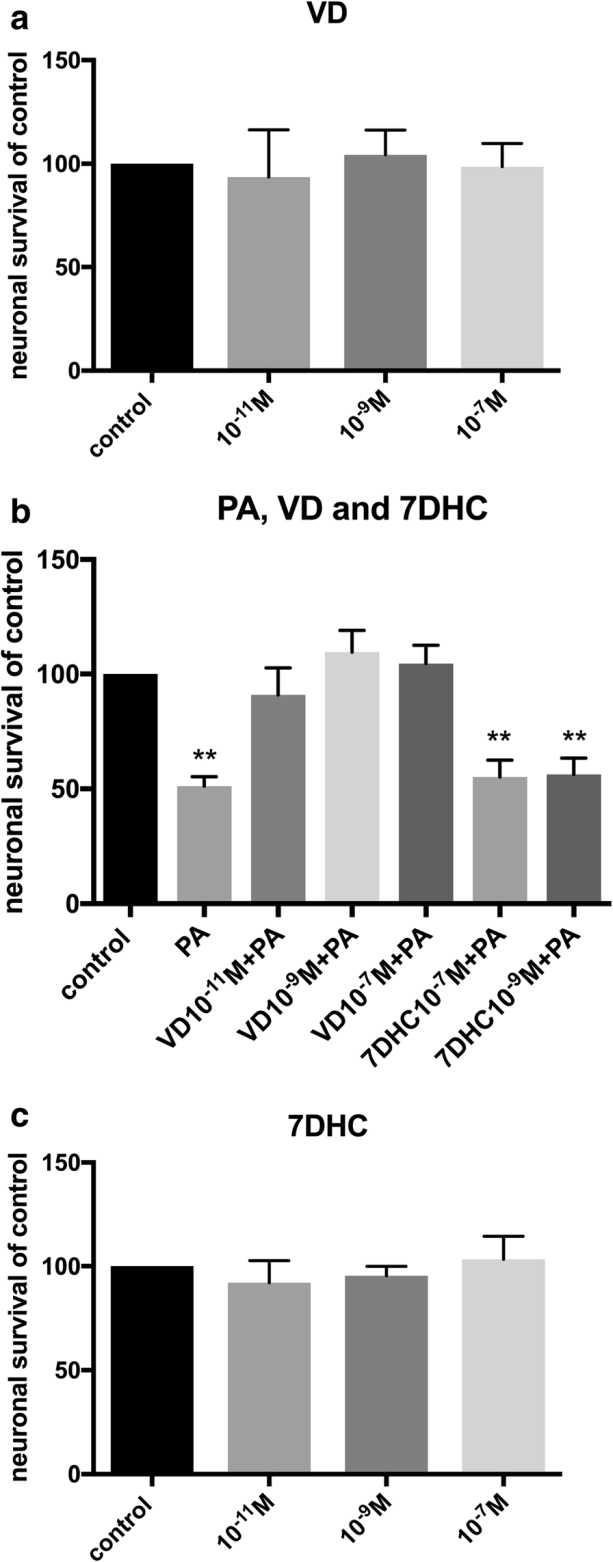
Effects of PA, VD and 7DHC in primary cultures of enteric neurons. **a**, **c** Supplementation with 1α,25-hydroxy-vitamin D3 (VD, 10^-11^-10^-7^M) or 7-dihydrocholesterol (7DHC 10^-11^-10^-7^M) had no effect on neuronal survival. **b** Cultures treated with palmitic acid (PA, 4x10^-4^M) induced enteric neuronal loss, supplementation with VD but not 7DHC prevented the PA induced loss. Untreated controls were run in parallel. Data presented as mean ± SEM, per treatment group, control *n*=6-12 per group, palmitic acid (PA) *n*=6, VD *n*=6-12, 7DHC, PA (4x10^-4^M) + VD (10^-11^-10^-7^M) *n*=6, PA (4x10^-4^M) +7DHC (10^-9^-10^-7^M) *n*=6, ** *p*<0.01

Primary cultures isolated from the longitudinal muscle layer of mouse small intestine contains myenteric ganglia, glia and smooth muscle cells, effects of PA exposure on these has previously been described [28]. Control wells displayed 2.2±0.1 neurons per mm^2^. Exposure to VD (10^-11^ – 10^-7^M) *per se* did not affect neuronal survival in cultures (Fig. 2a). VD serum concentration in mice are suggested to be in the 10^-10^M range [41]. Palmitic acid exposure alone induced a significant loss of cultured neurons (*p*<0.01, Fig. 2b), the loss was similar in magnitude to the previous described PA induced loss [28]. The combined exposure of PA (4x10^-4^M) and VD (10^-11^ – 10^-7^M) did not affect neuronal survival (Fig. 2b). Thus, the presence of VD (10^-11^ – 10^-7^M) abolished the previous described PA-induced neuronal loss. To test if the effect was due to the activated form of vitamin D (1α,25-hydroxy-vitamin D3, VD) cultures were exposed to the vitamin D precursor 7-dihydrocholesterol (7DHC). Exposure of 7DHC (10^-11^ – 10^-7^M) *per se* didn’t affect neuronal survival (Fig. 2c). Addition of 7DHC (10^-9^ – 10^-7^M) was unable to protect enteric neurons against the PA-induced neuronal loss (Fig. 2b).

### Localization of VDR and PDIA3 *in vivo* and *in vitro*

**Fig. 3:**
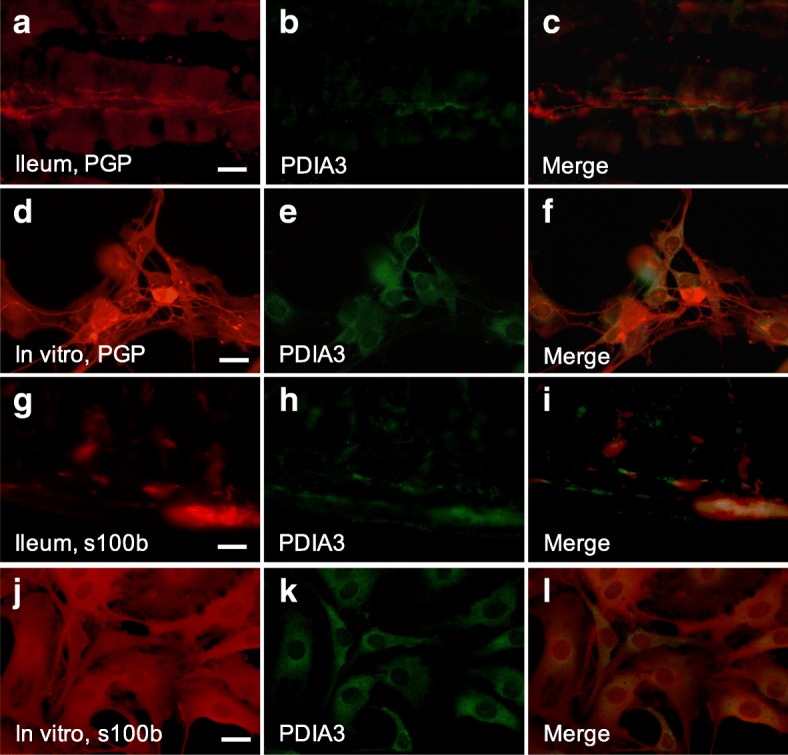
Localisation of PDIA3 in mouse and primary cultures of myenteric neurons. Representative micrographs of ileum of normal diet 1x vitamin D animals (**a**-**c**, **g**-**i**) and of primary cultures myenteric neurons (**d**-**f**, **j**-**l**). **a**-**f** Immunocytochemical staining of neuronal cell bodies and fibres using PGP9.5 in ileum (**a**) and culture (**d**) and PDIA3 positive fibres and cell bodies in ileum (**b**) and culture (**e**), merged images showing PDIA3 is not co-located with enteric neurons in ileum (**c**) and culture (**f**). **g**-**i** Immunocytochemical staining to enteric glia using S100β in ileum (**g**) and culture (**j**) and PDIA3 positive fibres and cell bodies in ileum (**h**) and culture (**k**), merged images show co-localisation of S100β and PDIA3, suggesting PDIA3 is expressed in enteric glia. Bars represent 20μm

Immunocytochemistry revealed VDR expression in mucosal epithelial cells of both small and large intestine while no VDR expression was detected in enteric neurons in vivo regardless of diet. In primary cultures of enteric neurons, no VDR expression was found in neurons. The alternative vitamin D receptor PDIA3 has been suggested to mediate the rapid effects of vitamin D in intestinal epithelial cells [15]. Immunocytochemistry revealed PDIA3 not to be expressed on enteric neurons (Fig. 3a-f) in vivo or in vitro, but to be expressed in intestinal glia in close connection to enteric neurons (Fig. 3g-i). This was evident in enteric neuronal cultures as well (Fig. 3j-l).

### In vitro inhibition of PDIA3 in primary enteric cultures

**Fig. 4:**
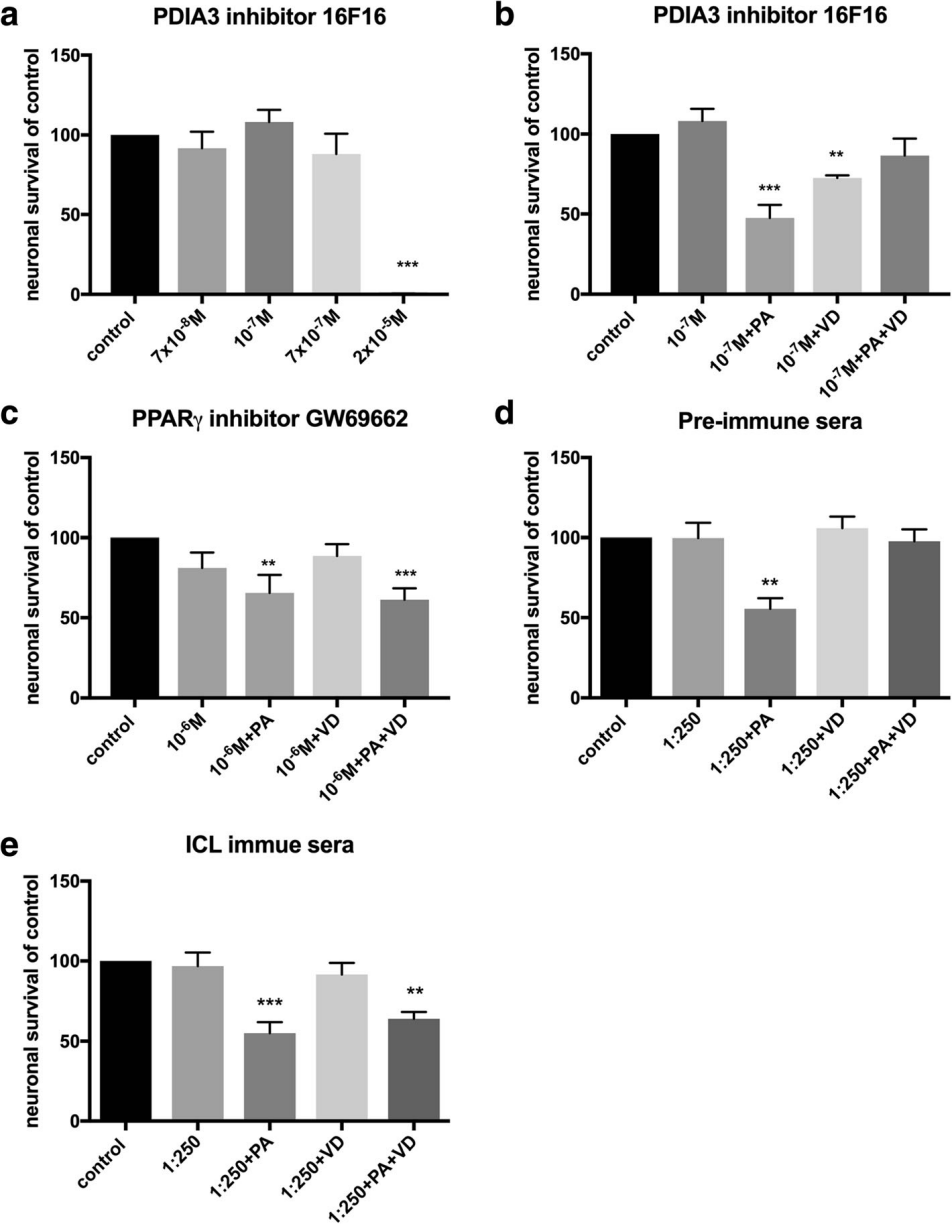
Effects of experimental treatment agent *per se* and on VD and VD+PA neuronal survival and PA-induced neuronal loss in primary cultures of myenteric neurons. **a** Supplementation with the protein disulphide isomerase family A member 3 (PDIA3) inhibitor 16F16 in the range 7x10^-8^M - 7x10^-7^M did not affect neuronal survival, but induced loss of all cells at 2x10^-5^ M. **b** Supplementation with the PDIA3 inhibitor (10^-7^M) had no effect on the palmitic acid (PA, 4x10^-4^M), the 1α,25-hydroxy-vitamin D3 (VD, 10^-7^M) or the PA+VD effects on neuronal survival. **c** Peroxisome proliferator-activated receptor gamma (PPARγ) inhibitor, GW69662 (10^-6^M) did not prevent the PA-induced neuronal loss but prevented the VD-induced prevention of the PA-induced loss. **d** Supplementation with pre-immune sera (1:250) had no effects on PA-, VD- or PA+VD-induced effects on neuronal survival. **e** Supplementation of isocitrate lyase (ICL) immune sera (1:250) did not prevent the PA-induced loss but did prevent the protective effects of VD on PA-induced loss. Untreated controls were run in parallel. Data presented as mean ± SEM, control *n*=6-18 per treatment agent, PDIA3 inhibitor 16F16 *n*=6-12, PPARγ inhibitor, GW69662 *n*=6, pre-immune sera *n*=6, ICL immune sera *n*=6, PDIA3 inhibitor (10^-7^M) + PA/VD/VD+PA *n*=6, PPARγ inhibitor (10^-6^M) + PA/VD/VD+PA *n*=6-12, pre immune sera (1:250) *n*=12, ICL immune sera (1:250) *n*=12-18, ** *p*< 0.01, *** *p*<0.001

To evaluate if the VD effect was acting indirect by way of PDIA3 activation in enteric glia, primary enteric cultures were exposed to 16F16, known to inhibit PDI’s including PDIA3 [38]. The inhibitor (7x10^-8^M - 2x10^-5^M) induced neuronal loss at concentrations above 7x10^-7^, with all cells in culture lost at 2x10^-5^ M (Fig. 4a). Supplementation with the PDIA3 inhibitor (10^-7^M) did not affect PA-induced neuronal loss in vitro nor did supplementation prevent the VD-mediated protection of the PA-induced neuronal loss. However, supplementation with the PDIA3 inhibitor (10^-7^M) significantly reduced neuronal survival in VD treated cultures (Fig. 4b).

### In vitro inhibition of PPARγ in primary enteric cultures

VDR is, like peroxisome proliferator-activated receptor (PPAR), a member of the nuclear hormone receptor family that form heterodimers with RXR. VDR and PPAR have interacting signaling pathways [42]. Primary enteric cultures were supplemented with the PPARγ inhibitor GW9662 (10^-6^M). The effect of GW9662 on cultured enteric neurons and glia, has been assessed in a previous study [28]. Supplementation of the PPARγ inhibitor did not prevent the PA induced neuronal loss, but did prevent the VD-mediated protection of the PA-induced neuronal loss (Fig. 4c).

### In vitro ICL antibody neutralization

Bacteria, fungi and plants use the glyoxylate cycle to bypass the CO_2_ producing step in the Krebs cycle, utilizing two enzymes, ICL and malate synthase. Exposing enteric neuronal cultures to pre-immune serum (1:250) did not affect neuronal survival *per se* or VD’s effect on PA-induced neuronal loss (Fig. 4d). Exposing cultures to ICL immune sera (1:250) did not affect neuronal survival *per se* nor the PA-induced neuronal loss. However, ICL immune sera supplementation prevented the protective effect of vitamin D on PA-induced neuronal loss (Fig. 4e).

## Discussion

Current study investigated the neuroprotective potential of vitamin D and VD on HFD- and PA-induced enteric neuronal loss. Vitamin D supplementation with 1.3 IU/g to 2.6 IU/g had no effect on either ND or HFD enteric neuronal survival. However, supplementation of vitamin D at 26 IU/g (20x) prevented the HFD-induced neuronal loss, without affecting ND neuronal survival. In vitro studies showed VD (10^-11^M-10^-7^M) to protect against PA-induced neuronal loss, this effect was due to the presence of VD as the biological inactive precursor 7HDC was unable to mimic this neuroprotective effect. Serum concentration of VD were not assessed in current study. However, it has been demonstrated that supplementation of 10x the normal vitamin D dose in mice does not lead to elevated serum VD levels (10^-10^M) nor induce hypercalcemia [41]. It is therefore not believed that supplementing with 20 times the normal dose would lead to toxicity. Further it has been shown that obesity is associated with lower vitamin D serum concentration, and patients should increase daily vitamin D intake [43].

### HFD- and PA-induced neurotoxicity

VD is a lipid soluble vitamin and adipocytes function as an important VD reservoir [44]. With the obesity epidemic and subsequent increase in type 2-diabetes [45], together with data suggesting that VD deficiency is a global health problem among all age groups [46], it is not surprising that the ability of VD to interfere with metabolic pathways have especially been studied in adipocytes and liver. In adipocytes, VD is involved in the regulation of differentiation, apoptosis, lipogenesis as well as acting as an anti-inflammatory agent [2, 47]. In liver, VD has experimentally been shown to be protective in HFD-induced steatosis. This is suggested to be through modulation of lipid metabolism, by increasing expression of enzymes and transcription factors involved in fatty acid oxidation i.e. carnitine-palmitoyl transferase 1a and 1b (CPT1a, b), pyruvate dehydrogenase kinase 4 (PDK4) and PPAR 1α. [1–3, 48] While studies have shown neurons capable of both mitochondrial and peroxisomal fatty acid oxidation [49], they are not believed to rely on lipids for energy production [50], this is seen by e.g. their low expression of CPT1a and CPT1b in mitochondrial membranes and their expression of CPT1c on endoplasmic reticulum (ER) membranes [51, 52]. HFD- and PA-induced enteric neuronal losses have been investigated previously [28, 29]. Using pharmacological agents interfering with intracellular lipid handling pathways it was suggested that PA-induced neuronal loss in vitro was through the culmination of multiple metabolic disturbances. This Includes excessive palmitoylcarnitine formation, exhausted carnitine stores, increased acetate-coenzyme A (-CoA) levels and energy depletion as well as increased levels of oxidative stress and membrane destabilization [28]. The ability of VD to interfere with these metabolic pathways was, in current study, tested in vitro.

### Receptor mediated VD effects

VD classically mediates its responses by binding to the nuclear VDR, leading to altered gene transcription, VDRs have also been found in plasma membranes, co-localized with caveolin 1, where they are suggested to mediate a fast response [53]. Upon activation VDR forms homodimers with RXR and these complexes modulate gene expression through binding to VDREs [13, 14]. In man more than 2700 genes have been identified to be regulated by VDR [14]. By immunocytochemistry VDR was not found in enteric neurons either in vivo or in vitro*.* It should, in this regard be noted that muscle cells (cardiac, skeletal and smooth) display positive VD mediated effects without clear evidence of the VDR being present [54]. It has been suggested that the non-genomic rapid effect of VD is through activation of the alternative VD receptor, PDIA3 [15]. PDIA3 has been ascribed pleiotropic effects, including reduction of cellular stress in neurons [55, 56], protection against autophagy induced apoptosis in beta cells [57], and protection against free fatty acid induced hepatosteatosis [58]. Immunocytochemistry showed PDIA3 to be found in enteric glia in vivo and in vitro. Enteric glia play important roles in the gut including acting as neuronal support and protection [59]. Inhibiting PDIA3 in vitro did not prevent the ability of VD to protect enteric neurons against PA-induced loss. This suggests that VD does not mediate its neuroprotective effect through activation of PDIA3 in enteric glia.

### Oxidative stress and membrane destabilization

VD mediates anti-inflammatory effects and has been shown to reduce HFD-induced increases in tumor necrosis factor α (TNFα) and pro-inflammatory cytokines such as interleukin (IL) -6 and lipid peroxidation products [60, 61]. Besides anti-inflammatory effects VD has also been shown to have anti-oxidative effects by expression of anti-oxidative enzymes such as superoxide dismutase (SOD), glutathione (GSH), and catalase [21, 22]. However, VD has also been shown, independent of receptor activation, to act as a membrane antioxidant by inhibiting lipid peroxidation. In the study, authors found the precursor 7HDC to be a more potent antioxidant than VD [19]. While current results showed 7HDC unable to prevent PA-induced neuronal loss, a direct anti-oxidative effect of VD in the protection against PA-induced loss cannot be entirely excluded.

### Energy metabolism

HFD disrupts the energy balance and pushes the system towards a more inflammatory and reactive oxygen species-rich phenotype. VD has been suggested to modulate energy pathways, including upregulating genes involved with fatty acid oxidation and anti-oxidation [3]. HFD has been shown to cause a reduced PPARγ expression and classical PPARγ targets including *CD36* in neurons [62], as well as in the small intestine [63]. In neurons PPARγ was suggested to act as neuronal lipid sensor, sensing and signaling to the central nervous system clues about the peripheral metabolic status [62]. In the intestinal epithelial cells, PPARγ is suggested to regulate barrier function and microbiome related inflammation [63]. We show that inhibition of PPARγ prevents the VD induced protection against the PA-induced neuronal loss in vitro, suggesting that high VD concentrations induce PPARγ activity.

### Alternative VD pathway

Plants, bacteria and nematodes have maintained the ability to synthesize glucose from lipids, using acetate produced from peroxisomal beta oxidation. This energy bypass is called the glyoxylate cycle. It is catalyzed by two enzymes isocitrate lyase (ICL) and malate synthase (MS) and is active in situations where environmental energy supply demands it [64]. In peroxisomes isocitrate is cleaved by ICL to form succinate and glyoxylate. Succinate enter the Krebs cycle and glyoxylate condenses with -CoA forming malate in a reaction catalyzed by MS. Malate can be converted into phosphoenolpyruvate by the enzyme phosphoenolpyruvate carboxykinase (PEPCK), and enter gluconeogenesis, thereby enabling lipid-derived carbon to be converted into glucose. The presence of this pathway in higher eukaryotes has been debated, including its presence and activity in peroxisomes of liver [65, 66], cartilage [67] and brown fat [68] as well as its absence. [69–71] Those suggesting its presence have shown that VD activate the glyoxylate or a glyoxylate-like cycle in liver and cartilage and stimulate the conversion of PA derived acetyl-CoA into glycogen [65, 67]. It is tempting to speculate that high VD exposure in enteric neurons is able to stimulate this pathway. Using immunoneutralization to inhibit ICL activity we showed that supplementing cultures with ICL antibodies, but not pre-immune sera, prevented the VD-induced protection against the PA-induced neuronal loss. Whether the ICL immune serum contains undetermined mediators capable of inhibiting the VD response has not been investigated in currently study and cannot be excluded. It is however, interesting to note that PPARγ is a known inducer of PEPCK, the enzyme enabling malate to enter gluconeogenesis. The induction of a glyoxylate or glyoxylate-like cycle has been suggested in hepatic glucose production [70, 72], where lipids increase hepatic gluconeogenesis. A working hypothesis in this respect could be that VD activates PAPRγ and glyoxylate cycle-like pathways, causing PA or PA-CoA shunting towards peroxisome degradation, gluconeogenesis and glucose production. In all creating a, for neurons, non-toxic metabolic intermediate that enables survival in a PA overload situation.

## Conclusion

In this study, we showed neuroprotective effects of VD against HFD- and PA-induced neuronal loss. The suggested pathway involves VD-induced activation of PPARγ which in turn improve neuronal peroxisome function and shuttling of PA-CoA. Suggesting that supplementation of vitamin D in the diet could protect enteric neurons against obesity-induced damage.

## Abbreviations

7DHC: 7-dihydrocholesterol
BSA: Bovine serum albumin
CDU: Collagen digestion units
-CoA: Acetyl- Coenzyme A
ENS: Enteric nervous system
ER: Endoplasmic reticulum
FBS: Fetal bovine serum
GSH: Glutathione
HBSS: Hank’s balanced salt solution
HFD: High fat diet
ICL: Isocitrate lyase
IL: Interleukin
IR: Immunoreactive
NBA: Neurobasal A
ND: Normal diet
PA: Palmitic acid
PBS: Phosphate buffered saline
PDI: Protein disulphide isomerase
PDIA3: Protein disulphide isomerase family A member 3
PDK4: Pyruvate dehydrogenase kinase 4
PEPCK: Phosphoenolpyruvate carboxykinase
PPARγ: Peroxisome proliferator-activated receptor gamma
ROS: Reactive oxygen species
RXR: Retinoid-X receptor
SOD: Superoxide dismutase
TNFα: Tumor necrosis factor alpha
VD: 1a,25-hydroxy-vitamin D3
VDR: VD receptor
VDREs: VD response elements

## References

[1] Yin Y, Yu Z, Xia M, Luo X, Lu X, Ling W (2012). Vitamin D attenuates high fat diet-induced hepatic steatosis in rats by modulating lipid metabolism. Eur J Clin Invest.

[2] Sergeev IN, Song Q (2014). High vitamin D and calcium intakes reduce diet-induced obesity in mice by increasing adipose tissue apoptosis. Mol Nutr Food Res.

[3] Marcotorchino J, Tourniaire F, Astier J, Karkeni E, Canault M, Amiot MJ, Bendahan D, Bernard M, Martin JC, Giannesini B (2014). Vitamin D protects against diet-induced obesity by enhancing fatty acid oxidation. J Nutr Biochem.

[4] Witham MD, Nadir MA, Struthers AD (2009). Effect of vitamin D on blood pressure: a systematic review and meta-analysis. J Hypertens.

[5] Vimaleswaran KS, Berry DJ, Lu C, Tikkanen E, Pilz S, Hiraki LT, Cooper JD, Dastani Z, Li R, Houston DK (2013). Causal relationship between obesity and vitamin D status: bi-directional Mendelian randomization analysis of multiple cohorts. PLoS Med.

[6] Danik JS, Manson JE (2012). Vitamin d and cardiovascular disease. Curr Treat Options Cardiovasc Med.

[7] Garcion E, Wion-Barbot N, Montero-Menei CN, Berger F, Wion D (2002). New clues about vitamin D functions in the nervous system. Trends Endocrinol Metab.

[8] Grimm Marcus, Mett Janine, Hartmann Tobias (2016). The Impact of Vitamin E and Other Fat-Soluble Vitamins on Alzheimer´s Disease. International Journal of Molecular Sciences.

[9] Wamberg L, Kampmann U, Stødkilde-Jørgensen H, Rejnmark L, Pedersen SB, Richelsen B (2013). Effects of vitamin D supplementation on body fat accumulation, inflammation, and metabolic risk factors in obese adults with low vitamin D levels - results from a randomized trial. Eur J Intern Med.

[10] Gallagher JC, Yalamanchili V, Smith LM (2013). The effect of vitamin D supplementation on serum 25OHD in thin and obese women. J Steroid Biochem Mol Biol.

[11] Eyles DW, Smith S, Kinobe R, Hewison M, McGrath JJ (2005). Distribution of the vitamin D receptor and 1 alpha-hydroxylase in human brain. J Chem Neuroanat.

[12] Overbergh L, Decallonne B, Valckx D, Verstuyf A, Depovere J, Laureys J, Rutgeerts O, Saint-Arnaud R, Bouillon R, Mathieu C (2000). Identification and immune regulation of 25-hydroxyvitamin D-1-alpha-hydroxylase in murine macrophages. Clin Exp Immunol.

[13] Schmidt DR, Mangelsdorf DJ (2008). Nuclear receptors of the enteric tract: guarding the frontier. Nutr Rev.

[14] Ramagopalan SV, Heger A, Berlanga AJ, Maugeri NJ, Lincoln MR, Burrell A, Handunnetthi L, Handel AE, Disanto G, Orton SM (2010). A ChIP-seq defined genome-wide map of vitamin D receptor binding: associations with disease and evolution. Genome Res.

[15] Nemere I, Hintze K (2008). Novel hormone “receptors”. J Cell Biochem.

[16] Doroudi M, Schwartz Z, Boyan BD (2012). Phospholipase A2 activating protein is required for 1α,25-dihydroxyvitamin D3 dependent rapid activation of protein kinase C via Pdia3. J Steroid Biochem Mol Biol.

[17] Wang Y, Chen J, Lee CS, Nizkorodov A, Riemenschneider K, Martin D, Hyzy S, Schwartz Z, Boyan BD (2010). Disruption of Pdia3 gene results in bone abnormality and affects 1alpha,25-dihydroxy-vitamin D3-induced rapid activation of PKC. J Steroid Biochem Mol Biol.

[18] Chen J, Olivares-Navarrete R, Wang Y, Herman TR, Boyan BD, Schwartz Z (2010). Protein-disulfide isomerase-associated 3 (Pdia3) mediates the membrane response to 1,25-dihydroxyvitamin D3 in osteoblasts. J Biol Chem.

[19] Wiseman H (1993). Vitamin D is a membrane antioxidant. Ability to inhibit iron-dependent lipid peroxidation in liposomes compared to cholesterol, ergosterol and tamoxifen and relevance to anticancer action. FEBS Lett.

[20] Farhangi MA, Nameni G, Hajiluian G, Mesgari-Abbasi M (2017). Cardiac tissue oxidative stress and inflammation after vitamin D administrations in high fat- diet induced obese rats. BMC Cardiovasc Disord.

[21] Noyan T, Balaharoğlu R, Kömüroğlu U (2005). The oxidant and antioxidant effects of 25-hydroxyvitamin D3 in liver, kidney and heart tissues of diabetic rats. Clin Exp Med.

[22] Bhat M, Ismail A (2015). Vitamin D treatment protects against and reverses oxidative stress induced muscle proteolysis. J Steroid Biochem Mol Biol.

[23] Singh VP, Bali A, Singh N, Jaggi AS (2014). Advanced glycation end products and diabetic complications. Korean J Physiol Pharmacol.

[24] Sandireddy R, Yerra VG, Areti A, Komirishetty P, Kumar A (2014). Neuroinflammation and oxidative stress in diabetic neuropathy: futuristic strategies based on these targets. Int J Endocrinol.

[25] Lv WS, Zhao WJ, Gong SL, Fang DD, Wang B, Fu ZJ, Yan SL, Wang YG (2015). Serum 25-hydroxyvitamin D levels and peripheral neuropathy in patients with type 2 diabetes: a systematic review and meta-analysis. J Endocrinol Invest.

[26] Boitard C, Cavaroc A, Sauvant J, Aubert A, Castanon N, Layé S, Ferreira G (2014). Impairment of hippocampal-dependent memory induced by juvenile high-fat diet intake is associated with enhanced hippocampal inflammation in rats. Brain Behav Immun.

[27] Kaczmarczyk MM, Machaj AS, Chiu GS, Lawson MA, Gainey SJ, York JM, Meling DD, Martin SA, Kwakwa KA, Newman AF (2013). Methylphenidate prevents high-fat diet (HFD)-induced learning/memory impairment in juvenile mice. Psychoneuroendocrinology.

[28] Voss U, Sand E, Olde B, Ekblad E (2013). Enteric neuropathy can be induced by high fat diet in vivo and palmitic acid exposure in vitro. PLoS One.

[29] Voss U, Turesson MF, Robaye B, Boeynaems JM, Olde B, Erlinge D, Ekblad E (2014). The enteric nervous system of P2Y13 receptor null mice is resistant against high-fat-diet- and palmitic-acid-induced neuronal loss. Purinergic Signal.

[30] Stenkamp-Strahm CM, Kappmeyer AJ, Schmalz JT, Gericke M, Balemba O (2013). High-fat diet ingestion correlates with neuropathy in the duodenum myenteric plexus of obese mice with symptoms of type 2 diabetes. Cell Tissue Res.

[31] Morin JP, Rodríguez-Durán LF, Guzmán-Ramos K, Perez-Cruz C, Ferreira G, Diaz-Cintra S, Pacheco-López G (2017). Palatable Hyper-Caloric Foods Impact on Neuronal Plasticity. Front Behav Neurosci.

[32] Eslick Guy D., Talley Nicholas J. (2016). Prevalence and relationship between gastrointestinal symptoms among individuals of different body mass index: A population-based study. Obesity Research & Clinical Practice.

[33] Wiltink J, Michal M, Wild PS, Zwiener I, Blettner M, Münzel T, Schulz A, Kirschner Y, Beutel ME (2013). Associations between depression and different measures of obesity (BMI, WC, WHtR, WHR). BMC Psychiatry.

[34] Furness JB, Callaghan BP, Rivera LR, Cho HJ (2014). The enteric nervous system and gastrointestinal innervation: integrated local and central control. Adv Exp Med Biol.

[35] Reeves PG, Nielsen FH, Fahey GC (1993). AIN-93 purified diets for laboratory rodents: final report of the American Institute of Nutrition ad hoc writing committee on the reformulation of the AIN-76A rodent diet. J Nutr.

[36] Sand E, Themner-Persson A, Ekblad E (2011). Corticotropin releasing factor-Distribution in rat intestine and role in neuroprotection. Regul Pept.

[37] Voss U, Sand E, Hellstrom PM, Ekblad E (2012). Glucagon-like peptides 1 and 2 and vasoactive intestinal peptide are neuroprotective on cultured and mast cell co-cultured rat myenteric neurons. BMC Gastroenterol.

[38] Hoffstrom BG, Kaplan A, Letso R, Schmid RS, Turmel GJ, Lo DC, Stockwell BR (2010). Inhibitors of protein disulfide isomerase suppress apoptosis induced by misfolded proteins. Nat Chem Biol.

[39] Voss U, Turesson MF, Robaye B, Boeynaems J-M, Olde B, Erlinge D, Ekblad E (2014). The enteric nervous system of P2Y13 _r_eceptor null mice is resistant against high fat diet- and palmitic acid-induced neuronal loss. Purinergic Signal.

[40] Nezami BG, Mwangi SM, Lee JE, Jeppsson S, Anitha M, Yarandi SS, Farris AB, Srinivasan S (2014). MicroRNA 375 Mediates Palmitate-Induced Enteric Neuronal Damage and High-Fat Diet-Induced Delayed Intestinal Transit in Mice. Gastroenterology.

[41] Ghaly S, Kaakoush NO, Lloyd F, McGonigle T, Mok D, Baird A, Klopcic B, Gordon L, Gorman S, Forest C (2018). High Dose Vitamin D supplementation alters faecal microbiome and predisposes mice to more severe colitis. Sci Rep.

[42] Matsuda S, Kitagishi Y (2013). Peroxisome proliferator-activated receptor and vitamin d receptor signaling pathways in cancer cells. Cancers (Basel).

[43] Ekwaru JP, Zwicker JD, Holick MF, Giovannucci E, Veugelers PJ (2014). The importance of body weight for the dose response relationship of oral vitamin D supplementation and serum 25-hydroxyvitamin D in healthy volunteers. PLoS One.

[44] Landrier JF, Marcotorchino J, Tourniaire F (2012). Lipophilic Micronutrients and Adipose Tissue Biology. Nutrients.

[45] Guariguata L, Whiting DR, Hambleton I, Beagley J, Linnenkamp U, Shaw JE (2014). Global estimates of diabetes prevalence for 2013 and projections for 2035. Diabetes Res Clin Pract.

[46] Palacios C, Gonzalez L (2014). Is vitamin D deficiency a major global public health problem?. J Steroid Biochem Mol Biol.

[47] Eugene C, Yangha K (2016). Vitamin D Decreases Adipocyte Lipid Storage and increases NAD-SIRT1 Pathway in 3T3-L1 Adipocytes. Nutrition.

[48] Yin Y, Yu ZW, Xia M, Luo XQ, Lu XF, Ling WH (2012). Vitamin D attenuates high fat diet-induced hepatic steatosis in rats by modulating lipid metabolism. Eur J Clin Invest.

[49] Berger J, Dorninger F, Forss-Petter S, Kunze M (2016). Peroxisomes in brain development and function. Biochim Biophys Acta.

[50] Romano A, Koczwara JB, Gallelli CA, Vergara D, Micioni Di Bonaventura MV, Gaetani S, Giudetti AM (2017). Fats for thoughts: An update on brain fatty acid metabolism. Int J Biochem Cell Biol.

[51] Lee Jieun, Wolfgang Michael J (2012). Metabolomic profiling reveals a role for CPT1c in neuronal oxidative metabolism. BMC Biochemistry.

[52] Sierra AY, Gratacos E, Carrasco P, Clotet J, Urena J, Serra D, Asins G, Hegardt FG, Casals N (2008). CPT1c is localized in endoplasmic reticulum of neurons and has carnitine palmitoyltransferase activity. J Biol Chem.

[53] Huhtakangas JA, Olivera CJ, Bishop JE, Zanello LP, Norman AW (2004). The vitamin D receptor is present in caveolae-enriched plasma membranes and binds 1 alpha,25(OH)2-vitamin D3 in vivo and in vitro. Mol Endocrinol.

[54] Wang Y, DeLuca HF. Is the vitamin d receptor found in muscle? BMC Biochem. 2012;13:23.

[55] Castillo V, Oñate M, Woehlbier U, Rozas P, Andreu C, Medinas D, Valdés P, Osorio F, Mercado G, Vidal RL (2015). Functional Role of the Disulfide Isomerase ERp57 in Axonal Regeneration. PLoS One.

[56] Pendyala G, Ninemire C, Fox HS (2012). Protective role for the disulfide isomerase PDIA3 in methamphetamine neurotoxicity. PLoS One.

[57] Yamamoto E, Uchida T, Abe H, Taka H, Fujimura T, Komiya K, Hara A, Ogihara T, Fujitani Y, Ueno T (2014). Increased expression of ERp57/GRP58 is protective against pancreatic beta cell death caused by autophagic failure. Biochem Biophys Res Commun.

[58] Zhang XQ, Pan Y, Yu CH, Xu CF, Xu L, Li YM, Chen WX (2015). PDIA3 Knockdown Exacerbates Free Fatty Acid-Induced Hepatocyte Steatosis and Apoptosis. PLoS One.

[59] Neunlist M, Rolli-Derkinderen M, Latorre R, Van Landeghem L, Coron E, Derkinderen P, De Giorgio R (2014). Enteric glial cells: recent developments and future directions. Gastroenterology.

[60] Erbaş O, Solmaz V, Aksoy D, Yavaşoğlu A, Sağcan M, Taşkıran D (2014). Cholecalciferol (vitamin D 3) improves cognitive dysfunction and reduces inflammation in a rat fatty liver model of metabolic syndrome. Life Sci.

[61] Kheder R, Hobkirk J, Saeed Z, Janus J, Carroll S, Browning MJ, Stover C (2017). Vitamin D3 supplementation of a high fat high sugar diet ameliorates prediabetic phenotype in female LDLR(-/-) and LDLR(+/+) mice. Immun Inflamm Dis.

[62] Liu C, Bookout AL, Lee S, Sun K, Jia L, Lee C, Udit S, Deng Y, Scherer PE, Mangelsdorf DJ (2014). PPARγ in vagal neurons regulates high-fat diet induced thermogenesis. Cell Metab.

[63] Tomas J, Mulet C, Saffarian A, Cavin JB, Ducroc R, Regnault B, Kun Tan C, Duszka K, Burcelin R, Wahli W (2016). High-fat diet modifies the PPAR-γ pathway leading to disruption of microbial and physiological ecosystem in murine small intestine. Proc Natl Acad Sci U S A.

[64] Dunn MF, Ramírez-Trujillo JA, Hernández-Lucas I (2009). Major roles of isocitrate lyase and malate synthase in bacterial and fungal pathogenesis. Microbiology.

[65] Davis WL, Matthews JL, Goodman DB (1989). Glyoxylate cycle in the rat liver: effect of vitamin D3 treatment. FASEB J.

[66] Davis WL, Goodman DB (1992). Evidence for the glyoxylate cycle in human liver. Anat Rec.

[67] Davis WL, Jones RG, Farmer GR, Cortinas E, Matthews JL, Goodman DB (1989). The glyoxylate cycle in rat epiphyseal cartilage: the effect of vitamin-D3 on the activity of the enzymes isocitrate lyase and malate synthase. Bone.

[68] Davis WL, Goodman DB, Crawford LA, Cooper OJ, Matthews JL (1990). Hibernation activates glyoxylate cycle and gluconeogenesis in black bear brown adipose tissue. Biochim Biophys Acta.

[69] Kondrashov FA, Koonin EV, Morgunov IG, Finogenova TV, Kondrashova MN (2006). Evolution of glyoxylate cycle enzymes in Metazoa: evidence of multiple horizontal transfer events and pseudogene formation. Biol Direct.

[70] Song S (2000). Can the glyoxylate pathway contribute to fat-induced hepatic insulin resistance?. Med Hypotheses.

[71] Popov VN, Igamberdiev AU, Schnarrenberger C, Volvenkin SV (1996). Induction of glyoxylate cycle enzymes in rat liver upon food starvation. FEBS Lett.

[72] Song S (2002). The role of increased liver triglyceride content: a culprit of diabetic hyperglycaemia?. Diabetes Metab Res Rev.

[73] Sand E, Voss U, Hammar O, Alm R, Nordin Fredrikson G, Ohlsson B, Ekblad E (2013). Gonadotropin-releasing hormone analog buserelin causes neuronal loss in rat gastrointestinal tract. Cell Tissue Res.

[74] Svensson D, Nebel D, Voss U, Ekblad E, Nilsson BO (2016). Vitamin D-induced up-regulation of human keratinocyte cathelicidin anti-microbial peptide expression involves retinoid X receptor α. Cell Tissue Res.

[75] Orchard GE (2000). Comparison of immunohistochemical labelling of melanocyte differentiation antibodies melan-A, tyrosinase and HMB 45 with NKIC3 and S100 protein in the evaluation of benign naevi and malignant melanoma. Histochem J.

